# Crocins Ameliorate Experimental Immune Checkpoint Inhibitor-Related Myocarditis by Targeting the Hpx/Nrf2/HO-1 Pathway

**DOI:** 10.3390/ijms27020911

**Published:** 2026-01-16

**Authors:** Jing Yan, Qingqing Cai, Yu Li, Yi Zhang, Ye Zhao, Fangbo Zhang, Huamin Zhang

**Affiliations:** 1 Institute of Chinese Materia Medica, China Academy of Chinese Medical Sciences, No. 16 Nanxiaojie, Dongzhimennei Ave, Beijing 100700, China; 2 Institute of Basic Theory for Chinese Medicine, China Academy of Chinese Medical Sciences, No. 16 Nanxiaojie, Dongzhimennei Ave, Beijing 100700, China

**Keywords:** crocins, *Crocus sativus* L., immune checkpoint inhibitor-related myocarditis, Hpx/Nrf2/HO-1 pathway

## Abstract

Immune checkpoint inhibitors (ICIs) for cancer therapy may induce immune-related adverse events including myocarditis, which occurs infrequently but carries a high mortality rate. Crocins are the active constituents derived from *Crocus sativus* L. (saffron), and have demonstrated various bioactivities including anti-tumor, anti-inflammation, antioxidation, anti-ischemia, anti-aging, and neuroprotective effects. This study established a subcutaneous xenotransplanted tumor model of human liver cancer in nude mice to better mimic ICI-related myocarditis. Animal experimental results revealed that crocins improved cardiac function, relieved myocardial damage and autoimmune response, and suppressed oxidative stress and inflammatory reaction. Quantitative proteomics and Western blotting verification confirmed that crocins ameliorated experimental ICI-related myocarditis by targeting the Hpx/Nrf2/HO-1 pathway. Molecular docking revealed that the best docking activities were demonstrated by crocin I–HO-1, crocin II–Hpx, and crocin III–Nrf2. These findings shed new light on the development of therapeutic strategies for treating ICI-related myocarditis and provided the fundamental basis for expanding the clinical application of crocins.

## 1. Introduction

Immune checkpoint inhibitors (ICIs) are novel treatments with demonstrated efficacy against many cancers [1]. They are monoclonal antibodies that block the host immune negative regulation receptors, such as cytotoxic T lymphocyte-associated protein 4 (CTLA-4), programmed cell death protein-1 (PD-1), and programmed death-ligand 1 (PD-L1). ICIs enhance the host immune activation against cancer cells and may thus induce immune-related adverse events. Immune checkpoint inhibitor (ICI)-associated toxicities can affect any organ, and myocarditis has emerged as an infrequent but lethal complication with an incidence of 0.04–1.14% and a mortality of 25–50% [2]. Although the precise mechanisms of ICI-induced myocarditis remain undefined, current reports indicate that a common antigen between tumor and myocardium shares high-frequency T cell receptor sequences [3]. The pathophysiology of cardiotoxicity is characterized by myocardial necrosis induced by active T cells infiltrating against muscle antigens, especially α-myosin [4]. Current drug treatments of ICI-induced myocarditis include corticosteroids and immunosuppressive therapies targeting T cells, which are not completely effective [5,6]. Moreover, high dosage of glucocorticoid causes serious side effects such as steroid diabetes [7]. Therefore, the development of new therapeutic drugs with fewer adverse reactions is urgently needed.

*Crocus sativus* L., known as saffron, contains >300 constituents, such as safranal, crocin, picrocrocin, and carotenoids, among which crocins are the main active components [8]. Crocins have extensive pharmacological effects, including anti-tumor, antitoxin, antioxidation, anti-inflammation, anti-depression, anti-ischemia, anti-aging, anti-hyperlipidemia, and antihypotension effects, and memory improvement [9]. Crocins may also exert cardioprotective effects in patients with breast cancer undergoing chemotherapy [10], and may protect cardiomyocytes against ICI-related myocarditis by inhibiting NLRP3-mediated pyroptosis via the NF-κB pathway [11].

In the current study, a subcutaneous xenotransplanted tumor model of human liver cancer in nude mice was constructed to closely simulate ICI-related myocarditis, and crocins were administered intragastrically for 14 consecutive days to evaluate therapeutic efficacy. Our results proved that crocin treatment enhanced cardiac function, alleviated myocardial damage and immune response, and suppressed oxidative stress and inflammatory reaction. Importantly, quantitative proteomics and molecular docking revealed hemopexin (Hpx) as the potential therapeutic target of crocins in ICI-related myocarditis. Experimental data revealed that crocin intervention relieved ICI-related myocarditis in a xenotransplanted tumor model of nude mice through regulating the Hpx/nuclear factor erythroid 2-related factor 2 (Nrf2)/heme oxygenase-1 (HO-1) pathway. Our findings elucidated the precise efficacy and potential mechanism of crocins in treatment of ICI-related myocarditis.

## 2. Results

### 2.1. High-Performance Liquid Chromatography (HPLC) Analysis of Crocins Sample

**Figure 1:**
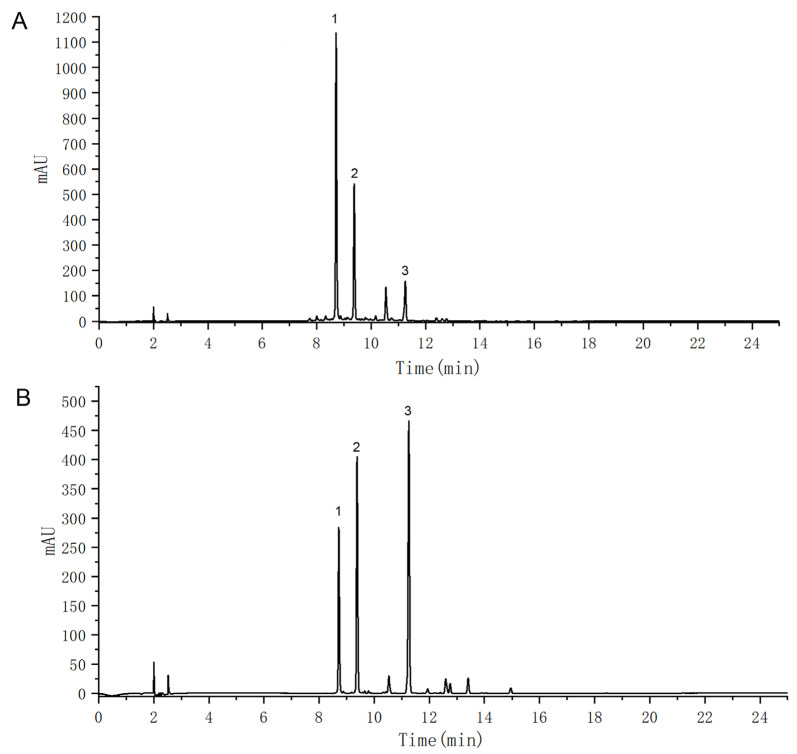
HPLC chromatograms of crocins samples (**A**) and compound standards (**B**). 1–crocin I; 2–crocin II; 3–crocin III.

HPLC analysis revealed that crocin I, II and III concentrations in the sample were 0.0652, 0.234, and 0.0069 g/g, respectively (Figure 1). The result further indicated that the quality of crocins used in this study was controlled and uniform.

### 2.2. Crocins Improved Cardiac Function in ICI-Related Myocarditis Mice

**Figure 2:**
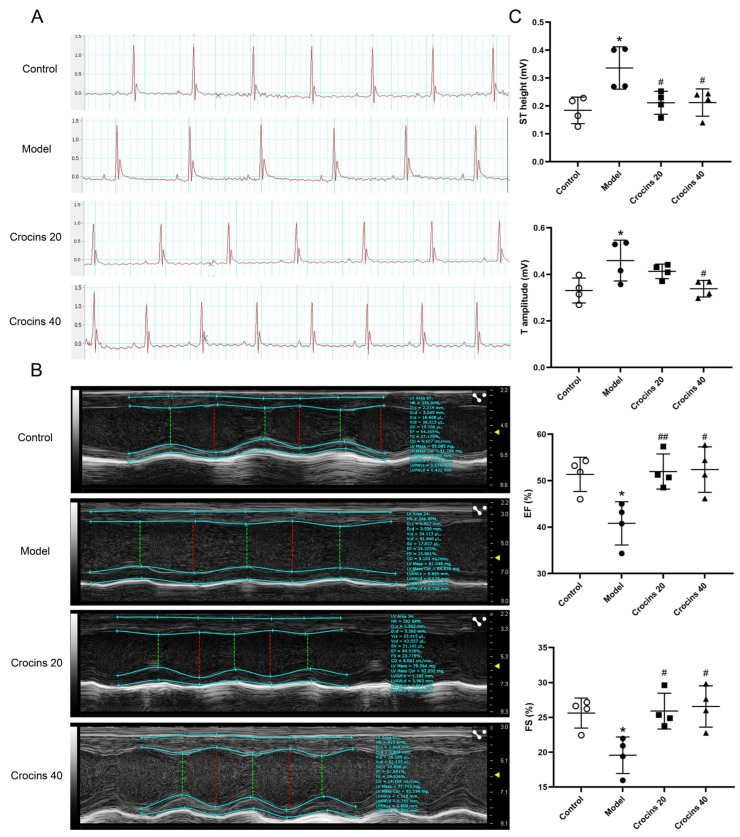
Crocins improved myocardial function of ICI-related myocarditis mice. (**A**) Representative electrocardiograms of mice from the different groups. (**B**) Typical transthoracic images of echocardiography. (**C**) Electrocardiographic and hemodynamic parameters including ST height, T amplitude, EF, and FS were evaluated (*n* = 4). Data are expressed as mean ± standard deviation (SD). (* *p* < 0.05 vs. control; ^#^ *p* < 0.05, ^##^ *p* < 0.01 vs. model).

Electrocardiography (ECG) showed that T amplitude (0.4589 ± 0.0875 mV) and ST height (0.3361 ± 0.0761 mV) in the model group increased significantly compared with the control group, indicating the existence of myocardial injury (Figure 2A,C) [12,13]. Compared with the model group, T amplitude in the high-dose crocins group and ST height in two crocins-treated groups significantly decreased. Echocardiography data showed that ejection fraction (EF; 40.81 ± 4.66%) and fraction shortening (FS; 19.56 ± 2.62 mL/min) in the model group were significantly lower than those in the control group (Figure 2B,C). However, EF and FS in the two crocins-treatment groups improved remarkably compared with the model group.

### 2.3. Crocins Mitigated Myocardial Damage and Immune Response in ICI-Related Myocarditis Mice

**Figure 3:**
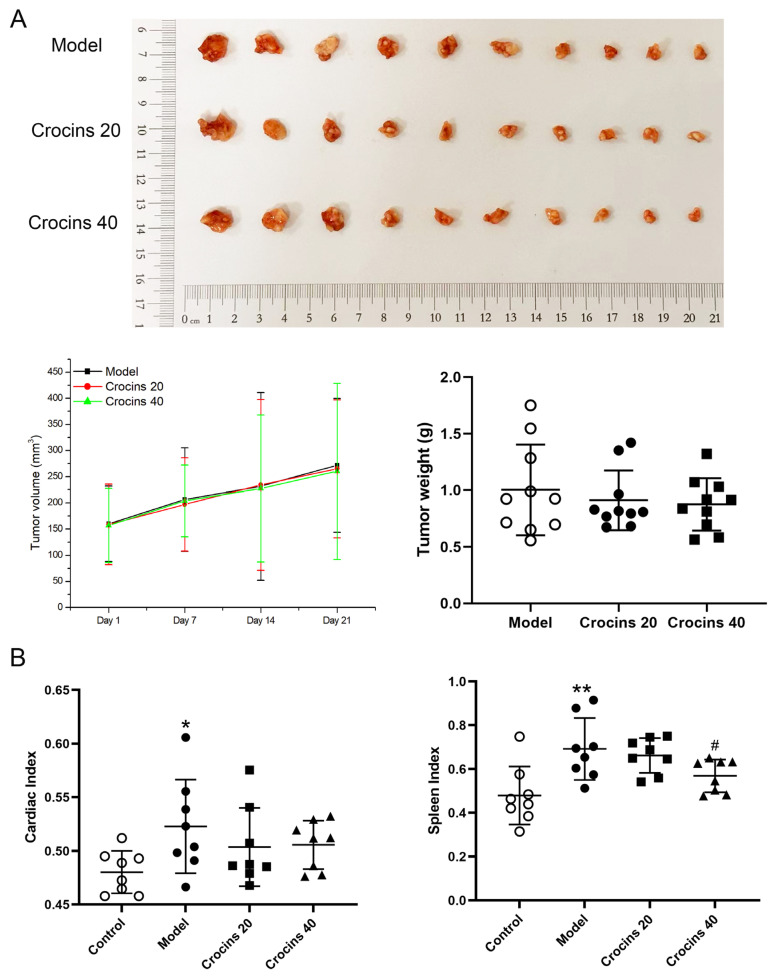

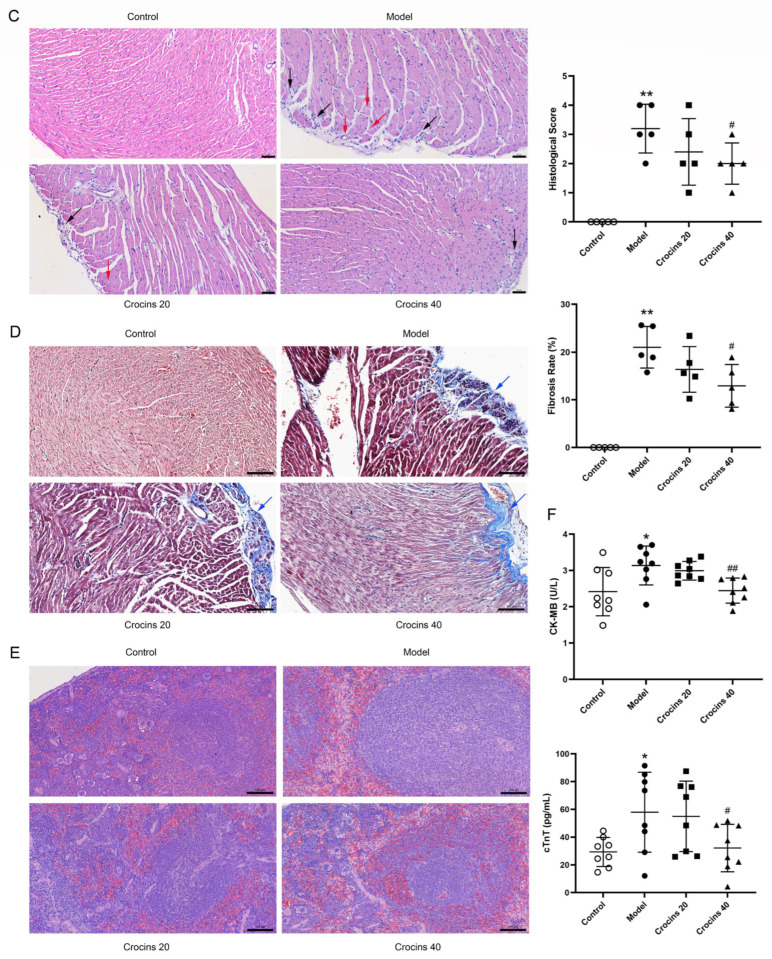
Crocins attenuated myocardial damage of ICI-related myocarditis mice. (**A**) Data of tumor volume and weight showed that crocins administration did not affect tumor growth in SK-Hep-1 xenografts. *n* = 10. (**B**) Heart and spleen indices (the ratio of cardiac or splenic mass/body weight, mg/g; *n* = 8. (**C**) Histopathological changes in the myocardium observed by HE staining, showing inflammatory infiltration (black arrow) and numerous necrotic cells (red arrow). Scale bar = 50 µm; *n* = 5. (**D**) Degree of myocardial tissue fibrosis observed by Masson staining, showing fibrotic tissue (collagen) deposition (blue arrow). Scale bar = 100 µm; *n* = 5. (**E**) Pathological features of the spleen observed by HE staining. Scale bar = 100 µm; *n* = 5. (**F**) Serum biomarkers including CK-MB and cTnT determined by enzyme-linked immunosorbent assay (*n* = 8). Data are expressed as mean ± SD. (* *p* < 0.05, ** *p* < 0.01 vs. control; ^#^ *p* < 0.05, ^##^ *p* < 0.01 vs. model).

Data showed that crocins did not result in any significant reduction in tumor volume and tumor weight compared with the model group (Figure 3A). These results suggested that crocins did not inhibit the tumor growth possibly duo to not reaching a sufficient anti-tumor concentration [14]. ICI-related myocarditis increased the heart and spleen indices (Figure 3B). However, the ratios of heart index and spleen indices significantly decreased in the high-dose crocins group.

Cardiac hematoxylin and eosin (HE) staining showed normal histology in the control group. In contrast, the model group showed massive inflammatory infiltration lesions, characterized by interstitial edema, cardiomyocyte swelling, and even myocardial necrosis (Figure 3C). However, these pathologic changes were alleviated in the crocins-treated groups, as revealed by histology grading. Masson staining showed numerous blue collagen fibers in the myocardial tissue of the model group (Figure 3D). The degree of cardiac fibrosis in the two crocins-treatment groups both decreased compared with the model group.

Spleen HE staining showed no histopathological changes in the control group (Figure 3E). In the model group, the white pulp area was enlarged, and the range of the germinal center increased, accompanied by an increase in lymphocytes. In contrast, the crocins-treated groups showed decreased white pulp and germinal center sizes with a lower number of lymphocytes.

Moreover, serum myocardial creatine kinase-MB isoenzyme (CK-MB) and cardiac troponin T (cTnT) levels in model mice notably increased (Figure 3F), which were significantly reduced by crocins treatment. Overall, our data illustrated that crocins at doses of 20 and 40 mg/kg effectively attenuated ICI-induced myocardial tissue damage.

### 2.4. Crocins Inhibited Oxidative Stress and Inflammatory Reaction in ICI-Related Myocarditis Mice

**Figure 4:**
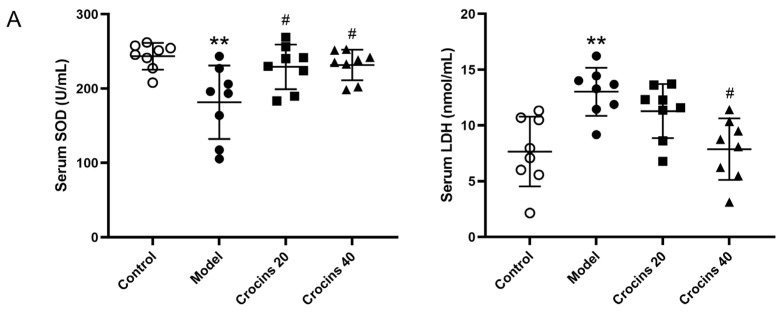

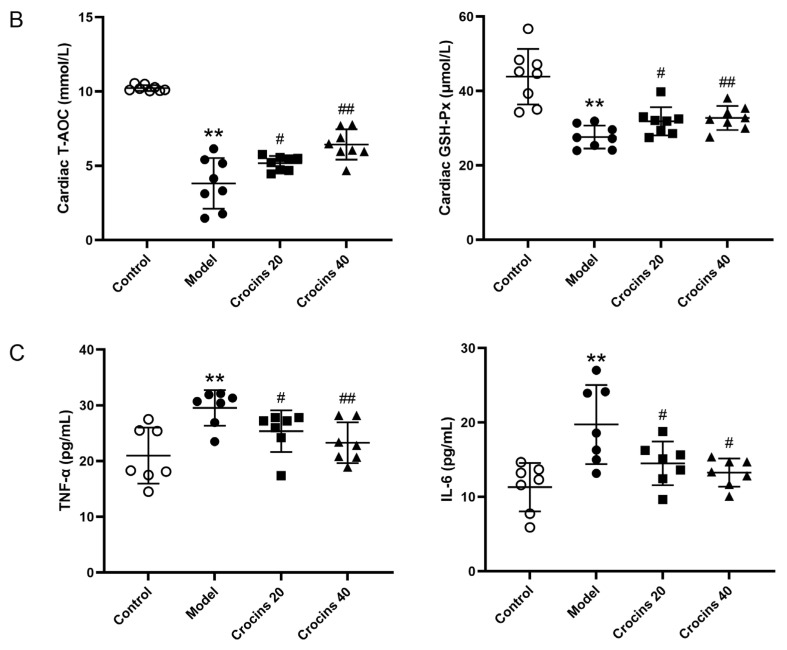
Crocins suppressed oxidative stress and inflammatory reaction in ICI-related myocarditis mice. (**A**) Oxidative stress-related molecules involving SOD and LDH in the serum examined using biochemical kits (*n* = 8). (**B**) Oxidative stress-related molecules including T-AOC and GSH-Px in the cardiac tissue determined using biochemical kits (*n* = 8). (**C**) Serum inflammatory cytokines detected using a Luminex technology-based multiplex system (*n* = 7). Data are expressed as mean ± SD. (** *p* < 0.01 vs. control; ^#^ *p* < 0.05, ^##^ *p* < 0.01 vs. model).

Compared with the control group, serum superoxide dismutase (SOD) activity decreased while serum lactate dehydrogenase (LDH) activity increased in the model group (Figure 4A). SOD activity increased in the two crocins-treated groups, while LDH activity decreased in the high-dose crocins group. The activities of total antioxidant capacity (T-AOC) and glutathione peroxidase (GSH-Px) in cardiac tissue of the model group were significantly decreased compared with the control group. Their activities were increased notably in the two crocins-treated groups in comparison with the model group (Figure 4B).

Furthermore, serum inflammatory cytokines, such as tumor necrosis factor (TNF)-α and interleukin (IL)-6, were higher in the model group than in the control group (Figure 4C). The serum levels of these two cytokines decreased in crocins-treated groups (20 and 40 mg/kg). These results demonstrated that crocins might exert protective effects in the myocardium by inhibiting oxidative stress and inflammatory response, as demonstrated in ICI-related myocarditis mice.

### 2.5. Proteomics Analysis Identified the Therapeutic Targets of Crocins in ICI-Related Myocarditis

**Figure 5:**
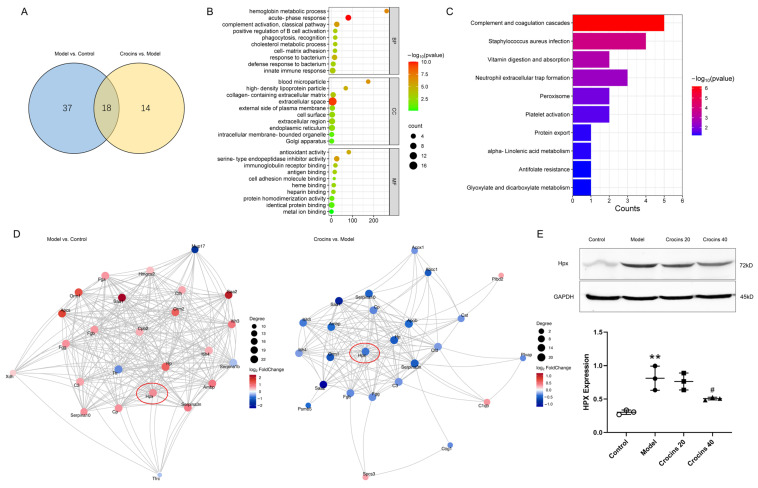
Functional analysis of crocins-regulated DEPs detected by tandem mass tag-based quantitative proteomics. (**A**) Venn diagram showing 18 overlapping targets identified through two types of comparison. (**B**) GO enrichment analysis of the DEPs based on the category of BP, CC, and MF. (**C**) KEGG enrichment analysis revealing the top 10 signaling pathways. (**D**) Two PPI network analysis constructed via two types of comparison. (**E**) Validation of crocins on Hpx expression in the cardiac tissue detected by Western blotting (*n* = 3). Data are expressed as mean ± SD. (** *p* < 0.01 vs. control; ^#^ *p* < 0.05 vs. model).

In total, 4191 proteins were identified from nine heart specimens. Compared with the control group, the model group had 36 upregulated and 19 downregulated differentially expressed proteins (DEPs). Compared with the model group, 7 and 25 proteins in the high-dose crocins group were significantly increased and decreased, respectively. Two types of comparisons revealed 18 overlapping DEPs (Figure 5A).

Gene ontology (GO) enrichment analysis revealed that 309 biological processes (BPs), 406 cellular components (CCs), and 403 molecular functions (MFs) were obtained in the comparison between the model and crocins-treated groups. The BP term was mainly correlated with the acute-phase response and innate immune responses (Figure 5B). Kyoto Encyclopedia of Genes and Genomes (KEGG) enrichment analysis revealed that the involved signaling pathways were principally associated with complement and coagulation cascades, *Staphylococcus aureus* infection, vitamin digestion and absorption, neutrophil extracellular trap formation, peroxisome, platelet activation, protein export, alpha-linolenic acid metabolism, glyoxylate and dicarboxylate metabolism, and antifolate resistance (Figure 5C).

Two protein–protein interaction (PPI) networks were established to explore the potential therapeutic targets of crocins against ICI-related myocarditis (Figure 5D). Hemopexin (Hpx), a common protein, was selected as the probable target and then verified by Western blotting. As expected, Hpx expression increased in the model group (0.81 ± 0.18) compared with the control group (0.30 ± 0.03). The expression significantly decreased in the high-dose crocins groups (0.51 ± 0.02; Figure 5E). The pattern of Hpx expression was consistent with that observed in the quantitative proteomics analysis.

### 2.6. Crocins Exerted Protective Effects Against ICI-Related Myocarditis by Regulating Hpx/Nrf2/HO-1 Pathway

**Figure 6:**
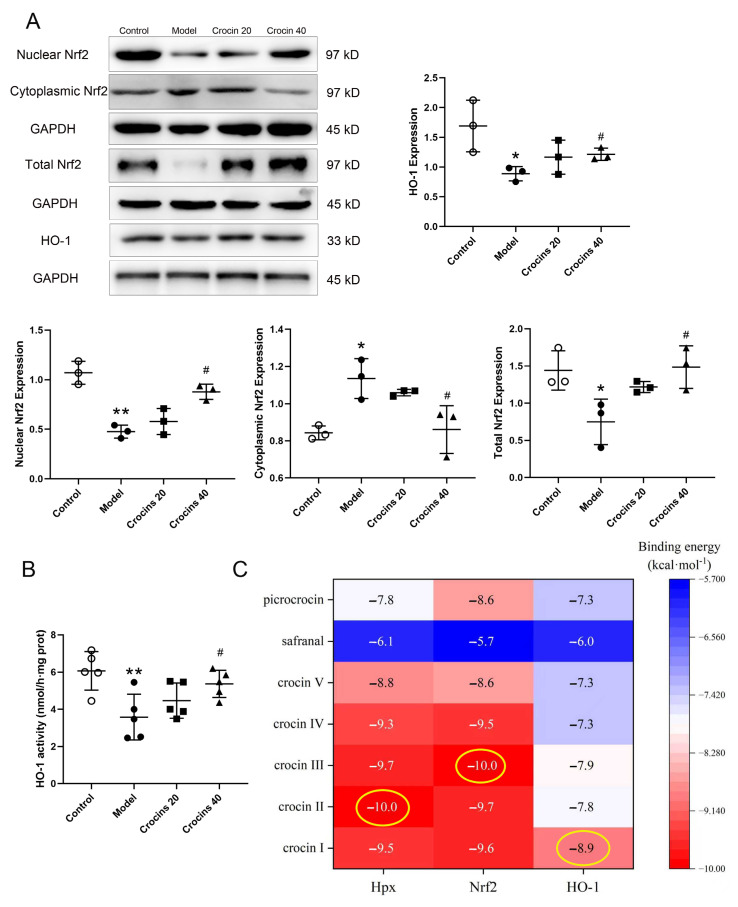

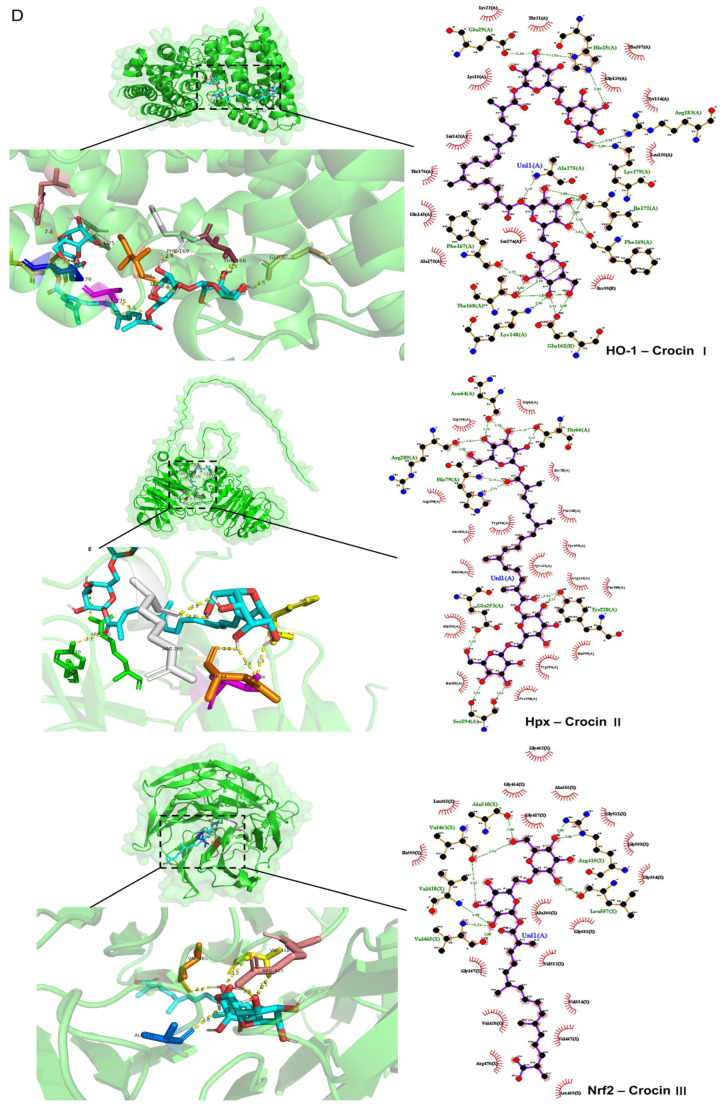
Crocins exerted protective effects against ICI-related myocarditis through the Nrf2/HO-1 pathway. (**A**) Expressions of nuclear Nrf2, cytoplasmic Nrf2, total Nrf2, and HO-1 detected by Western blotting (*n* = 3). (**B**) Cardiac HO-1 activity determined by the colorimetric detection kit (n = 5). Data are expressed as mean ± SD. (* *p* < 0.05 vs. control; ** *p* < 0.01 vs. control; ^#^ *p* < 0.05 vs. model). (**C**) Heat map showing molecular docking scores of the compounds and central targets. (**D**) Molecular docking 2D and 3D diagrams of the components and central targets as follows: HO-1–crocin I, Hpx–crocin II, and Nrf2–crocin III.

Nrf2/HO-1 pathway activation can significantly reduce inflammatory responses in various pathological events, such as cancer, cardiovascular diseases, and renal ischemia–reperfusion injury [15,16]. The link between Hpx and the Nrf2/HO-1 signaling pathway was then examined. ICI-related myocarditis reduced the expressions of nuclear Nrf2 (0.47 ± 0.06), total Nrf2 (0.74 ± 0.30), and HO-1 (0.88 ± 0.12) compared with the control group (nuclear Nrf2, 1.06 ± 0.11; total Nrf2, 1.44 ± 0.26; HO-1, 1.69 ± 0.43; Figure 6A). Crocins treatment activated the expressions of nuclear Nrf2, total Nrf2, and HO-1 in the cardiac tissue of model mice. Nuclear Nrf2, total Nrf2, and HO-1 expressions in the high-dose crocins group were (0.87 ± 0.07), (1.48 ± 0.28), and (1.21 ± 0.10), respectively, which were significantly upregulated compared with those in the model group. However, the expression of cytoplasmic Nrf2 in the model group (1.34 ± 0.10) was increased compared with the control group (0.84 ± 0.03), and it was downregulated in the high-dose crocins group (0.86 ± 0.12) compared with the model group. Moreover, cardiac HO-1 activity of the model group (3.57 ± 1.23 nmol/h·mg prot) was significantly declined in comparison with the control group (6.06 ± 1.03 nmol/h·mg prot, Figure 6B). HO-1 activity of the high-dose crocins group (5.36 ± 0.72 nmol/h·mg prot) was elevated notably in comparison with the model group. These findings demonstrated that the cardioprotective effect of crocins was partially due to the activation of the Hpx/Nrf2/HO-1 signaling pathway.

Crocins are a group of carotenoid compounds isolated from *Crocus sativus* L., which contains seven different natural crocin analogs, namely, crocins I to VII [17,18], as well as other active compounds, such as picrocrocin and safranal [19]. According to the literature, these nine chemical components of crocins were selected for molecular docking with the three core targets: Hpx, Nrf2, and HO-1. Affinity < −7.0 kcal·mol^−1^ is regarded as strong docking activity [20]. The best docking activities were screened in HO-1–crocin I (−8.9), Hpx–crocin II (−10.0), and Nrf2–crocin III (−10.0) (Figure 6C,D). Furthermore, crocin I, II, and III showed good binding affinities with the corresponding targets, indicating their potential therapeutic effects in ICI-related myocarditis.

## 3. Discussion

Fatal myocarditis occurs because immune check-point receptors are expressed in human cardiomyocytes, which are targeted by ICIs while eliminating cancer cells [21,22]. Patients with ICI-related myocarditis may develop arrhythmia and even heart failure [23], although clinical manifestations vary according to disease severity and individual differences. Tachycardia results from the damage to myocardial cells, while bradycardia develops from the infiltration of lymphocytes of the sinoatrial or atrioventricular node. These conditions cause prominent ECG abnormalities, such as ST segment elevation, prolonged PR interval, and low QRS voltage [24]. The echocardiographic changes include abnormal wall motion, segmental motor dysfunction, and diastolic dysfunction [25]. Preclinical studies have shown that the left ventricle EF and global radial strain were significantly reduced in transplanted melanoma mice treated with anti-PD-1 antibodies [26]. Our data showed that crocins shortened the T amplitude and ST height and increased EF and FS, thereby enhancing cardiac function in the ICI-related myocarditis mouse model.

Serum CK-MB and cTnT are common clinical myocardial injury markers. CK-MB is mainly distributed in myocardial cells. When the myocardium is damaged, the serum CK-MB level increases with high specificity. Approximately 94–97% of patients with myocarditis exhibit elevated CK-MB levels [27]. However, no correlation has been established between patients with myocarditis and elevated serum troponin levels [28]. Furthermore, cTnT is mostly used to diagnose myocardial infarction [29]. Crocins reduced serum CK-MB and cTnT levels in ICI-related myocarditis model mice. Cardiac HE and Masson staining further confirmed that crocins can alleviate myocardial damage caused by the ICI.

Oxidative stress is involved in the pathogenesis of many cardiovascular diseases. Excessive oxidative stress can damage DNA, proteins, and other biological molecules [30]. During the process of inflammation and oxidative stress, LDH is produced abundantly via anaerobic glycolysis to provide energy. SOD is an antioxidant substance that exerts myocardial protection by eliminating oxygen free radicals. Increasing evidence indicates that inflammation and autoimmunity are critical in the pathogenesis of autoimmune myocarditis [31]. Under inflammatory conditions, macrophages activated fibroblasts by releasing pro-inflammatory cytokines, such as TGF-β and TNF-α [32]. In autoimmune myocarditis model rats, the inflammatory response increases the expression of type I and III collagen [33], and initiates reparative fibrosis, ultimately leading to cardiac fibrosis [34]. Saffron extract can suppress myocardial necrosis and edema in isoproterenol-induced myocardial infarction model rats [35]. In particular, crocin inhibits MMP and may directly bind to MMP-2, thereby reducing extracellular matrix degradation [36]. Autoimmune myocarditis model mice exhibited extremely dilated hearts and enlarged spleens due to abnormal spontaneous autoimmunity [37]. Reduced heart and spleen indices together with spleen HE staining indicated that crocins suppressed the autoimmune response caused by the ICI. Our data further demonstrated that crocins alleviated myocardial injury by inhibiting oxidative stress and the inflammatory response.

Hpx is a circulating glycoprotein mainly expressed in the liver. It has the highest binding affinity to heme, an iron-containing pro-oxidant and pro-inflammatory molecule. This binding prevents heme-bound iron loss and inhibits heme-mediated inflammation and oxidative stress [38]. Evidence has demonstrated that induction of circulating Hpx increases anthracycline cardiac toxicity in patients and in mice [39]. However, aspirin eugenol ester could reverse the upregulation trend of six proteins such as Hpx, and then have a positive effect on a rat thrombosis model [40]. Meanwhile, clinical colorectal cancer patients often have high Hpx levels, which predicts poor prognosis in older patients [41]. Therefore, Hpx increase or decrease associated with disease protection is not completely clear. Our research revealed that under the experimental background of coexists with liver tumor and ICI-related myocarditis, crocins decreased the expression level of Hpx and thus exerted protective effects.

HO-1 is a heat shock protein and a rate-limiting enzyme that catalyzes excessive heme degradation [42]. HO-1 is involved in several physiological functions including antioxidation, anti-inflammation, and anti-apoptosis, and it can promote angiogenesis, suppress cell differentiation, and induce mitochondrial autophagy [43]. HO-1 is a downstream factor regulated by Nrf2, which is a transcription factor that is activated by high oxidative stress to regulate many antioxidant and detoxification genes [44]. Nrf2 improves cardiac function and modulates the release of pro-inflammatory cytokines in animal models of major cardiovascular diseases [45]. The Nrf2/HO-1 axis is highly correlated with the intracellular defense mechanism against oxidative stress, and it is a common therapeutic target of cardiovascular diseases, such as myocardial infarction, viral myocarditis, and myocardial ischemia–reperfusion injury. When activated, Nrf2 is translocated to the nucleus, and binds to DNA via antioxidant response elements, thereby upregulating HO-1 expression to reduce oxidative stress. Based on the critical role of the Nrf2/HO-1 signaling pathway in cardiac diseases, we performed proteomics screening that identified Hpx as a key link between this pathway, the pathogenesis of ICI-related myocarditis, and the therapeutic action of crocins. Molecular docking further revealed the high binding affinity of the primary crocins (I–III) for HO-1, Hpx, and Nrf2. Our experimental data further indicate that crocins suppressed oxidative stress and the inflammatory immune response by regulating the Hpx/Nrf2/HO-1 pathway, thereby mitigating myocardial injury.

The present study also has several key limitations. First, the construction method of an animal model is limited by the availability of experimental materials. This research established a complex animal model combing subcutaneous xenotransplanted tumor with ICI-related myocarditis, so as to better mimic the clinical condition of side effects induced by ICI therapy. Our preliminary experiment confirmed that only pembrolizumab (human anti-PD-1 monoclonal antibody) intraperitoneally injected in the xenotransplanted tumor nude mice for 14 consecutive days could not induce ICI-related myocarditis, which was mainly caused by myosin immunization in our study. Second, a single sex (male nude mice) and a single dosing regimen restricted the accuracy of efficacy evaluation. Third, future research should employ silenced models to definitively investigate the causal role of the Hpx/Nrf2/HO-1 signaling pathway in ICI-related myocarditis. Finally, our findings provide a mechanistic foundation to sufficiently support our conclusions, and extensive validation of functional experiments may be further conducted.

## 4. Materials and Methods

### 4.1. Experimental Animals, Cells and Drugs

Male BALB/c-nude mice aged 6–8 week were procured from Beijing HFK Bioscience Co., Ltd., Beijing, China. The mice were housed in a specific pathogen-free environment under a 12 h light/dark cycle at 22 °C. All the in vivo animal experiment procedures were approved by the Animal Care and Use Committee of the China Academy of Chinese Medical Sciences. Human hepatocarcinoma cell line SK-Hep-1 was purchased from Wuhan Pricella Biotechnology Co., Ltd. (Wuhan, China). Pembrolizumab (purity ≥ 99.0%; 25.1 mg/mL in phosphate buffer) was purchased from Abmole Bioscience Inc. (Houston, TX, USA). Crocins were kindly provided by Reyoung Pharmaceutical Co., Ltd. (Zibo, China).

### 4.2. Chemical Analysis of Crocins Sample

To ensure drug sample quality and stability, the content of the three main crocin components (crocin I, II, and III) in crocins were analyzed by HPLC performed on a Vanquish Core HPLC System (Thermo Fisher Scientific, Waltham, MA, USA) equipped with an autosampler (VC-A12-A-02), variable wavelength detector (VC-D40-A-01), and column compartment (VC-C10-A-03). The mobile phase consisted of 0.1% formic acid (A) and acetonitrile (B) with a flow rate of 1.0 mL/min. The detection wavelength was 440 nm, and the injection volume was 5 μL. The optimized gradient program was as follows: from 0 to 20 min (5%–95% B); 20–23 min (95%–95% B); and 23–25 min (95%–5% B). The column temperature was maintained at 25 °C. The standard substances of the three compounds were all obtained from Jiangxi Baicaoyuan Biotechnology Co., Ltd. (Nachang, China; purity ≥ 98% for all).

### 4.3. Animal Model Construction and Group Design

SK-Hep-1 cells were cultivated in high-glucose Dulbecco’s Modified Eagle Medium (Gibico, Grand Island, CA, USA) supplemented with 10% fetal bovine serum (Tianhang, Hangzhou, Zhejiang, China), and penicillin-streptomycin (Solarbio, Beijing, China) in a humidified incubator (Sanyo, Tokyo, Japan) with 5% CO_2_ at 37 °C. When grown to nearly 80–90% confluence, cells were digested with 0.25% trypsin (Solarbio, Beijing, China) and then centrifuged at 800 rpm/min for 5 min. The cells were then resuspended in an appropriate amount of culture medium and adjusted to a density of 5 × 10^7^ cells/mL. The mouse tumor model was established via the subcutaneous injection of SK-Hep-1 cells (0.2 mL). Tumor sites were inspected daily. The tumor length (L) and width (W) were measured with a caliper, and tumor volume was calculated using the formula L × (W)^2^/2. The tumor growth inhibition rate was calculated according to the following formula: (tumor volume of the control group-tumor volume of the experimental group)/tumor volume of the control group × 100%.

**Figure 7:**
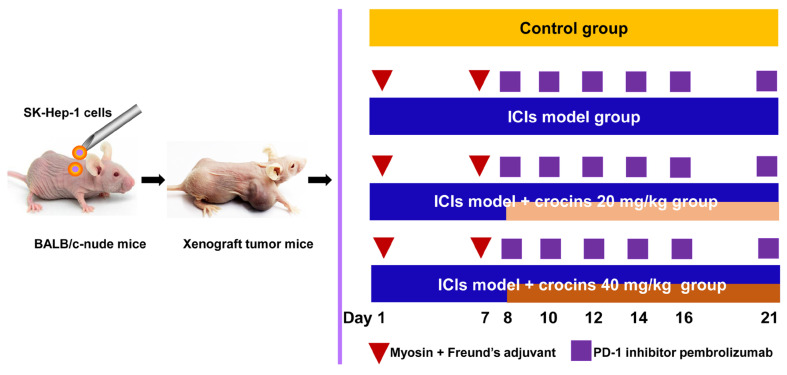
Schematic diagram of ICI-related myocarditis induction and administration times of PD-1 inhibitor pembrolizumab and crocins. The grouping day was counted as the first day. Myosin and Freund’s adjuvant were subcutaneously injected on day 1 and 7. Pembrolizumab was intraperitoneally injected on day 8, 10, 12, 14, 16, and 21.

When tumor volume had reached approximately 150 mm^3^, the successfully constructed mice models were randomly separated into four groups (n = 10 per group): control, ICI-related myocarditis model, ICI-related myocarditis + low-dose crocins (20 mg/kg/day), and ICI-related myocarditis + high-dose crocins (40 mg/kg/day) groups (Figure 7). The grouping day was counted as the first day of the animal experiment. The ICI-related myocarditis model in nude mice was constructed as reported [11]. Except for the control group, all the mice were immunized with a subcutaneous injection of 133 μg calcium-activated myosin (from porcine heart, Sigma-Aldrich, St. Louis, MO, USA) diluted with complete Freund’s adjuvant (Sigma-Aldrich, St. Louis, MO, USA) on days 1 and 7. From day 8, mice were administrated an intraperitoneal injection of pembrolizumab every 2 days at a dose of 5 mg/kg/day for a total of 5 injections to induce ICI-related myocarditis. The mice in the two crocins-treated groups were administered with crocins by gavage once per day for 14 consecutive days starting from day 8. The control group was subcutaneously injected with complete Freund’s adjuvant, intraperitoneally injected with phosphate buffer, and orally given deionized water, all at the same volumes as the treatments and on the same days.

### 4.4. Electrocardiograph and Echocardiography

After 14 days of crocins administration, three mice were randomly selected from each group for ECG and echocardiography examinations with a Vevo3100 high-resolution ultrasonography imaging system (FUJIFLIM VisualSonics, Toronto, ON, Canada). Mice were anesthetized using 3% isoffurane (RWD, Shenzhen, Guangdong, China). When the breathing of the mouse stabilized, three ECGs were obtained using needle electrodes inserted subcutaneously in each forelimb and hindlimb. ECG parameters, such as heart rate, RR interval, PR interval, P duration, QRS interval, QT interval, corrected QT, JT interval, T_peak_–T_end_ interval, P amplitude, Q amplitude, R amplitude, S amplitude, T amplitude, and ST height, were recorded and analyzed using the digital acquisition and analysis system. The recorded echocardiographic parameters included systolic left ventricular anterior wall, diastolic left ventricular anterior wall, systolic left ventricular posterior wall thickness, diastolic left ventricular posterior wall thickness, left ventricular end-diastolic diameter, and left ventricular end-systolic diameter. Cardiac hemodynamic data on EF, cardiac output, stroke volume, and FS were assessed offline using Vevo LAB analytical software (version 5.5.0).

### 4.5. Organ Index and Histological Examination

Following ECG and echocardiography, all the mice were weighed, and then intraperitoneally anesthetized with pentobarbital sodium (Sigma-Aldrich, St. Louis, MO, USA) for blood sample collection from the orbital venous plexus. The heart and spleen were quickly removed and weighed. The organ index was calculated as the ratio of the organ mass to the body weight (mg/g).

After weighing, myocardial and spleen tissues were fixed in 4% paraformaldehyde (Solarbio, Beijing, China) and embedded in the paraffin wax. Sections were cut 5 µm thick and then stained. HE and Masson’s trichrome staining were performed to assess myocardial inflammation and fibrosis, respectively. Myocarditis was evaluated as the presence of inflammatory infiltration. The slides were observed under a light microscope (Olympus, Tokyo, Japan), and scored as follows: 0, no inflammation; 1, <5% inflammatory infiltration; 2, 5–10% inflammatory infiltration; 3, 10–20% inflammatory infiltration; 4, >20% inflammatory infiltration [46]. Cardiac fibrosis was determined by measuring the collagen volume fraction as the proportion of the blue fibrotic area to the total area [47]. The degree of myocardial infiltration and fibrosis was determined blindly by two independent investigators in a blinded manner. Five microscopic fields were randomly selected for each section to be photographed. Image J software (version 1.51j8) was applied to calculate the ratio of the area of inflammatory cell infiltration in each field to the area of the whole field, and also distinguish the blue color area from the total area for judging collagen fibers.

### 4.6. Serum Biomarkers and Inflammatory Cytokines

Serum biomarkers of myocardial injury, including CK-MB and cTnT, were measured using enzyme-linked immunosorbent assay kits (Inselisa, Huangshi, China). Serum levels of oxidative stress-related molecules, such as serum LDH and SOD, cardiac T-AOC, and GSH-Px were determined using the corresponding biochemical kits from Nanjing Jiancheng Bioengineering Institute (Nanjing, China).

Serum inflammatory cytokines were detected using a Luminex-based multiplex assay system (BioPlex, BioRad, Hercules, CA, USA). In total, 23 inflammatory cytokines were monitored using the multiplex immunoassay kit, including IL-1α, IL-1β, IL-2, IL-4, IL-5, IL-6, IL-7, IL-8 (GRO/KC), IL-10, IL-12 (p70), IL-13, IL-17A, IL-18, TNF-α, interferon-γ, monocyte chemoattractant protein-1, macrophage inflammatory protein (MIP)-1α, MIP-3α, granulocyte colony-stimulating factor (CSF), macrophage-CSF, granulocyte-macrophage-CSF, vascular endothelial growth factor, and regulated upon activation, normal T cell expressed and secreted.

### 4.7. Tandem Mass Tag-Based Quantitative Proteomics

Nine myocardial tissue replicates (n = 3 per group) were selected from the control, model, and high-dose crocins groups. The total proteins were extracted using lysis solution (Solarbio, Beijing, China) and proteinase inhibitors (Sigma-Aldrich, St. Louis, MO, USA), and the lysate was centrifuged at 13,000× *g* for 15 min (4 °C). The supernatant was collected, and the concentration of the protein sample was qualified using a bicinchoninic acid kit (BCA; Pierce, Rockford, IL, USA). Subsequently, the trypsin-digested peptides were labeled with 10-plex TMT reagents (Thermo Fisher Scientific, Waltham, MA, USA) according to the manufacturer’s instructions. The signed protein samples were fractionated using an Ultimate 3000 HPLC system (Thermo Fisher Scientific, Waltham, MA, USA), after which the fractional proteins were vacuum-dried. The peptides were dissolved in 0.1% formic acid and then detected using an EASY-nLC1200 Orbitrap Elite system (Thermo Fisher Scientific, Waltham, MA, USA). GO and KEGG analyses were performed using the DAVID database (https://david.ncifcrf.gov/ (accessed on 27 September 2023)). DEPs were considered statistically significant at a *p*-value of < 0.05 and at a fold change ≥1.2 or ≤0.8 [48]. Statistical differences were evaluated by Benjamini–Hochberg-adjusted hypergeometric testing (corrected *p* < 0.05) to control false discovery rates.

### 4.8. Western Blotting

Myocardial tissues were lysed with ice-cold radioimmunoprecipitation assay buffer and protease inhibitors (Solarbio, Beijing, China) under ultrasonication (Xinzhi, Ningbo, Zhejiamg, China) and centrifuged at 13,000× *g* for 15 min (4 °C). The concentration of the protein sample was determined using a BCA protein assay kit (Pierce, Rockford, IL, USA). Nuclear and cytoplasmic Nrf2 proteins were obtained by the NE-PER™ nuclear and cytoplasmic extraction reagents (Thermo Fisher Scientific, Waltham, MA, USA). The protein sample (40 μg) was separated by sodium dodecyl sulfate–polyacrylamide gel electrophoresis and then transferred onto a polyvinylidene membrane (Merck Millipore, Billerica, MA, USA). The membrane was blocked with a diluted primary antibody Hpx (1:1000, cat. no. MA5-35760, Thermo Fisher Scientific), HO-1 (1:1000, cat. no. #43966, Cell Signaling Technology, Danvers, MA, USA), and Nrf2 (1:1000, cat. no. #12721, Cell Signaling Technology, Danvers, MA, USA) overnight at 4 °C. The protein band was exposed with an enhanced chemiluminescence agent (Merck Millipore, Billerica, MA, USA). GAPDH (1:1000, cat. no. #2118, Cell Signaling Technology, Danvers, MA, USA) served as an internal reference.

### 4.9. Cardiac HO-1 Activity Assay

The cardiac HO-1 enzymatic activity was measured by the colorimetric detection kit purchased from Shanghai Haling Biotechnology Co., Ltd., Shanghai, China. The detection principle is based on the generating determination of Ferene-S and carbon monoxide through NADPH mediation. The unit of HO-1 activity (nmol/h·mg prot) is defined as the amount of enzyme required to yield 1 nM biliverdin per hour at the condition of 37 °C and pH 7.4. All the experimental procedures were strictly operated in accordance with the instructions of the test kit. The concentration of the tissue sample was qualified using a BCA protein quantification kit (Pierce, Rockford, IL, USA). The absorbance of Ferene-S and biliverdin was determined using a microplate reader (Molecular Devices, Sunnyvale, CA, USA) at 464 nm and 530 nm, respectively.

### 4.10. Molecular Docking

Seven compounds from crocins (crocin I, crocin II, crocin III, crocin IV, crocin V, safranal and picrocrocin) were obtained through documentation retrieval. The three-dimensional (3D) structure were retrieved from the PubChem database (http://pubchem.ncbi.nlm.nih.gov/ (accessed on 21 February 2025)). The 3D crystal structures of the three molecular targets Hpx, Nrf2, and HO-1 were downloaded from the Worldwide Protein Data Bank database (https://www.rcsb.org/ (accessed on 21 February 2025)). Molecular docking of the receptor and ligand as well as free-binding energies were performed using the online CB-Dock2 platform (https://cadd.labshare.cn/cb-dock2/php/index.php (accessed on 25 February 2025)). The docking results were visualized using the PyMol (https://pymol.org/2/ (accessed on 28 February 2025)) and LigPlot^+^ (https://www.ebi.ac.uk/thornton-srv/software/LigPlus/ (accessed on 28 February 2025)) software applications.

### 4.11. Statistical Analysis

All data were analyzed using SPSS 20.0 software (IBM, Armonk, NY, USA), and expressed as mean ± SD. Statistical significance was determined using one-way ANOVA followed by Tukey’s post hoc multiple comparison test. A *p*-value < 0.05 was considered statistically significant. The normality of data distribution for quantitative analysis was verified using the Shapiro–Wilk test before parametric statistical tests. All statistical analyses were performed and visualized using GraphPad Prism version 9.0 (GraphPad Software, Boston, MA, USA).

## 5. Conclusions

**Figure 8:**
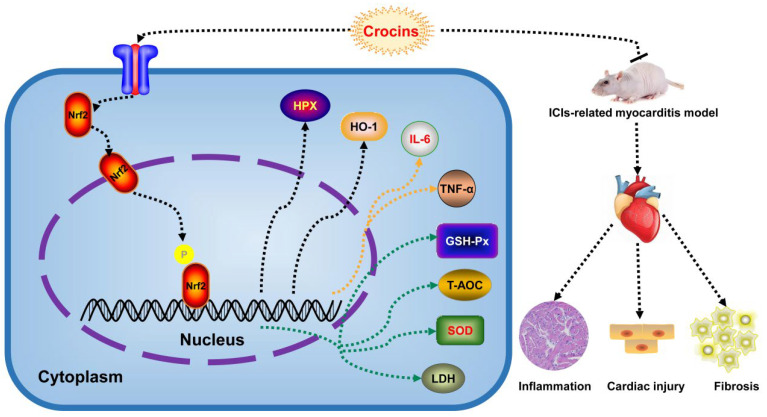
Schematic description of crocins relieving myocardial injury against ICI-related myocarditis via the Hpx/Nrf2/HO-1 pathway.

Our study has demonstrated that crocins exhibit cardioprotective effects against ICI-related myocarditis with extremely typical characteristics of multi-component and multi-target intervention (Figure 8). The animal experiment shows that crocins improve cardiac function, ameliorate myocardial injury and autoimmune response, and suppress oxidative stress inflammatory reaction through regulating the Hpx/Nrf2/HO-1 signaling pathway. Molecular docking data further confirm that crocin I, crocin II, and crocin III are potentially the main active compounds of crocins exerting therapeutic effects against ICI-related myocarditis. Our study provides valuable insights into facilitating the development of new therapeutic strategies for treating ICI-related myocarditis.

## Data Availability

TMT proteomics data have been submitted to the iProX database (https://www.iprox.cn/ (accessed on 28 December 2025)), and the accession number is PXD072469. Other datasets analyzed during the current study may be available upon reasonable request to the corresponding author.

## Abbreviations

The following abbreviations are used in this manuscript:

ICIs	Immune checkpoint inhibitors
CTLA-4	Cytotoxic T lymphocyte-associated protein 4
PD-1	Programmed cell death protein-1
PD-L1	Programmed death-ligand 1
ICI	Immune checkpoint inhibitor
Hpx	Hemopexin
Nrf2	Nuclear factor erythroid 2-related factor 2
HO-1	Heme oxygenase-1
HPLC	High-performance liquid chromatography
ECG	Electrocardiography
EF	Ejection fraction
FS	Fraction shortening
SD	standard deviation
HE	Hematoxylin and eosin
CK-MB	Creatine kinase-MB isoenzyme
cTnT	Cardiac troponin T
SOD	Superoxide dismutase
T-AOC	Total antioxidant capacity
GSH-Px	Glutathione peroxidase
LDH	Lactate dehydrogenase
TNF	Tumor necrosis factor
IL	Interleukin
DEPs	Differentially expressed proteins
GO	Gene ontology
BP	Biological process
CC	Cellular component
MF	Molecular function
KEGG	Kyoto Encyclopedia of Genes and Genomes
PPI	Protein–protein interaction
MIP	Macrophage inflammatory protein
CSF	Colony-stimulating factor
